# Texture Phenotypes of Fiber-Enriched Extruded Snacks Revealed by Mechanical–Acoustic Analysis, Tribology, and Sensory Mapping

**DOI:** 10.3390/foods15040758

**Published:** 2026-02-19

**Authors:** Aunchalee Aussanasuwannakul, Hataichanok Kantrong

**Affiliations:** 1 Department of Food Chemistry and Physics, Institute of Food Research and Product Development, Kasetsart University, Bangkok 10903, Thailand; 2 Department of Food Processing and Preservation, Institute of Food Research and Product Development, Kasetsart University, Bangkok 10903, Thailand

**Keywords:** extruded snacks, instrumental texture, crispness, Acoustic Envelope Detector (AED), tribology, oral processing, sensory analysis, multivariate analysis, okara, dietary fiber

## Abstract

Texture perception in extruded snacks is commonly evaluated using force-based measurements, although crispness-related oral sensations arise from fracture, sound emission, and lubrication during mastication. This study developed a mechanistically grounded framework for texture characterization of fiber-enriched extruded snacks by integrating instrumental and sensory analyses within an oral-processing context. Extruded snack samples containing soybean residue (okara; 0%, 29%, and 40%) and commercial benchmarks were evaluated using synchronized mechanical–acoustic testing (five-blade Allo-Kramer shear and three-point bending tests), oral tribology, and sensory evaluation combining intensity rating and ranking. Increasing okara content shifted fracture behavior from brittle, sound-emitting failure toward damped, progressive deformation with approximately 3–5-fold lower acoustic envelope amplitudes and smoother force–time profiles. These changes corresponded to lower perceived Crunchiness and Sound Intensity, reflecting diminished crispness-related perception, and higher Hardness and Grittiness/Coarseness attributes (increases of ~25–45%). Oral tribology revealed cohesive, poorly lubricated boli for okara-rich snacks, requiring higher entrainment parameters (*Uη*_0_ ≈ 1.0 × 10^5^–3.5 × 10^5^) to transition from boundary to mixed lubrication compared with commercial benchmarks (*Uη*_0_ ≈ 7.0 × 10^4^–2.0 × 10^5^). Convergent multivariate analyses established instrumentally defined texture phenotypes that translate mechanical–acoustic and tribological signatures into sensory-interpretable texture categories, providing a practical framework for discriminating and optimizing nutritionally enhanced extruded snack products.

## 1. Introduction

Crispness is one of the most desirable attributes in dry snack products, strongly shaping consumer expectations of freshness, quality, and eating enjoyment. It arises from rapid brittle fracture events that generate simultaneous tactile and auditory cues—sharp force drops, multiple breakages, and high-frequency sound bursts—making crispness an inherently multisensory perception rather than a purely mechanical one [1,2,3]. Structural characteristics such as porosity, cell-wall thickness, moisture content, and expansion ratio play a central role in determining crisp fracture behavior and sound generation [4,5], and recent studies continue to confirm these relationships in expanded or puffed grain-based snacks (e.g., Refs. [6,7]). Consequently, the food industry continues to seek reliable instrumental approaches—including mechanical, acoustic, and tribological measurements—to objectively quantify and engineer crisp textures in a repeatable manner [8,9], with newer work emphasizing multisensory, structure–texture integration [10,11].

In parallel with these technical challenges, the snack sector has seen growing interest in fiber-rich, upcycled ingredients to meet sustainability targets and enhance nutritional value. Soybean residue (okara), a major by-product of soymilk and tofu production, is particularly attractive due to its high dietary fiber, protein content, and water-holding capacity. However, these same attributes introduce pronounced textural challenges: okara incorporation is widely associated with reduced expansion, increased bulk density, and changes in fracture behavior and mouthfeel in dried snack matrices [8,12,13], reflecting texture–nutrient trade-offs also observed in recent pulse- and legume-enriched extruded snacks [14,15]. As a result, achieving desirable crispness in okara-enriched snacks remains difficult, and structure–texture relationships are often poorly predicted by conventional force-based instrumental measurements.

Our previous studies illustrate both the opportunity and complexity of using okara in crispy food systems. In extruded snacks, increasing okara levels (0–50%) led to reduced radial expansion, increased density, diminished crispness perception, and altered lubrication behavior, with approximately 19% okara identified as a practical compromise between texture quality and consumer acceptance [16]. Similarly, in gluten-free crispy waffles, okara flour addition (10–40%) improved batter elasticity and nutritional profile but increased hardness and reduced spread ratio, with 30% okara yielding the highest overall sensory acceptance [17]. These findings underscore okara’s dual role: while nutritionally and environmentally advantageous, its fiber-rich nature fundamentally alters fracture mechanics and oral texture in ways that are not fully captured by traditional texture metrics—an issue echoed in recent evaluations of other high-fiber extrudates [18].

Recent advances in crispness research offer new possibilities for addressing this limitation. Mechanical–acoustic fracture profiling, jaggedness and rupture-pattern analysis, energy-based crispness descriptors, and tribology-informed interpretations of oral processing have all contributed to a more nuanced understanding of how structural failure translates into sensory perception [3,19,20]. Nevertheless, these state-of-the-art approaches have been applied primarily to conventional commercial snacks or fresh horticultural products. Recent tribology and mouthfeel reviews further emphasize the importance of integrating lubrication behavior into texture research, particularly for dry and particulate foods [21,22].

The present study addresses this gap by integrating mechanical–acoustic characterization, oral tribology, and sensory mapping to examine how okara incorporation modifies crispness and oral-processing behavior in extruded snacks. By jointly analyzing rupture-force signatures, acoustic burst patterns, lubrication regimes, and semi-trained panel sensory descriptors, this work aims to establish mechanistic markers of crispness tailored specifically to fiber-rich, plant-based, and upcycled snack matrices. In doing so, it provides industry-relevant insight into how sustainable ingredients such as okara can be incorporated into extruded snacks while retaining desirable crispy texture and eating quality.

## 2. Materials and Methods

### 2.1. Materials

**Figure 1:**
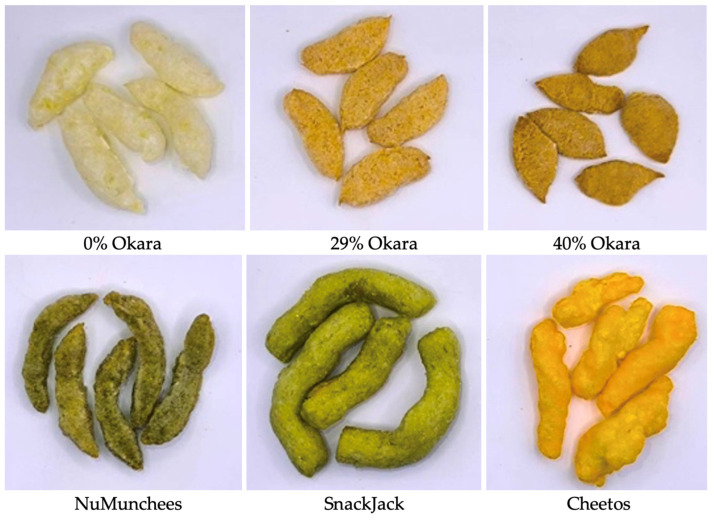
Snack samples evaluated in this study: okara-based prototype extrudates (**top row**; 0%, 29%, and 40% okara, *w*/*w*) and commercial benchmarks (**bottom row**; NuMunchees, SnackJack, and Cheetos). All products were tested as received after equilibration to room temperature.

Six snack samples, comprising three prototype formulations and three commercial benchmarks, were used in this study (Figure 1). The prototype snacks were produced at the Institute of Food Research and Product Development (IFRPD), Kasetsart University, and incorporated graded levels of soybean residue (okara) at 0%, 29%, and 40% (*w*/*w*). These levels were selected based on prior findings that okara markedly influences expansion, crispness, hardness, and lubrication behavior during mastication, while sensory acceptability remains high for formulations containing up to approximately 40% okara. Accordingly, the 29% and 40% okara levels were selected to represent moderate and high substitution levels within the practical formulation range for extruded snacks, enabling systematic evaluation of texture changes under nutritionally relevant enrichment conditions. The 0% formulation served as a starch-rich baseline, whereas the 29% and 40% formulations represented moderate and high fiber–protein systems, respectively. Their proximate composition reflects this gradient, with dietary fiber increasing from 4.3% in the control to 20.4% in the 40% okara snack (Table 1).

To ensure meaningful comparison across a broad range of commercial extruded snack structures, three benchmark products were deliberately selected to span distinct compositional and textural archetypes commonly encountered in the snack market. To provide external reference points for texture, mouthfeel, and lubrication behavior, three commercial snacks were selected based on their dominant ingredient profiles and contrasting sensory archetypes. NuMunchees, a mung-bean-based extrudate with 20% protein and moderate dietary fiber, served as a protein-rich legume benchmark. SnackJack, a green pea snack containing 25.8% fat, provided a high-fat, crunchy comparator with distinctly greasy mouthfeel. Cheetos, a corn-based commercial extrudate with 40% fat, represented a starch-rich, airy, and brittle texture commonly associated with puffed corn snacks. These benchmarks span a wide compositional space—starch-rich, protein-rich, legume-based, and high-fat systems—thereby enabling robust comparative analyses across acoustic–mechanical fracture behavior, oral tribology, and sensory characterization using Flash Profile. All products were stored in their original packaging and equilibrated to room temperature prior to testing.

In addition to the snack samples, all chemicals used for tribological testing were of analytical grade. Artificial saliva was prepared following the formulation described by [23] to simulate both the enzymatic and lubricating functions of human saliva. The solution contained sodium chloride (0.117 g/L), potassium chloride (0.149 g/L), and sodium bicarbonate (2.100 g/L) (Merck, Darmstadt, Germany); mucin from porcine stomach (Type II, Sigma-Aldrich, St. Louis, MO, USA; M2378) at 1.000 g/L; and α-amylase from *Bacillus* sp. (Sigma-Aldrich, St. Louis, MO, USA; A6814) at 0.050 g/L (approximately 4000 U/L enzymatic activity). All components were dissolved in LC–MS-grade double-distilled water and the artificial saliva was freshly prepared prior to each experiment. The final solution was equilibrated to 37 °C before use.

### 2.2. Formulation and Sample Preparation

The prototype snacks were formulated using okara flour, mung bean flour, rice flour, and corn grit. Fresh okara obtained from IFRPD’s pilot-scale soymilk production line was tray-dried at 100 °C for four hours to reduce moisture to below 10% and subsequently milled to a particle size of 30–50 mesh. All dry ingredients were weighed according to their respective formulations and homogenized in a planetary mixer for five minutes to ensure uniform distribution prior to extrusion. Extrusion was carried out using an intermeshing co-rotating twin-screw extruder (Hermann Berstorff ZE25×33D, Hermann Berstorff GmbH, Hanover, Germany), equipped with a square die opening (15 mm height × 20 mm length), under the same thermal, mechanical, and moisture conditions previously described by [16] to ensure process comparability. The barrel temperature profile across the seven heating zones (from feeder to die) was set at 37, 55, 121, 132, 164, and 111 °C, with a screw speed of 400 rpm and feed moisture adjusted to 15–17%. Material was fed continuously through a volumetric twin-screw feeder, and melt temperatures remained between 152–155 °C.

No other extrusion parameters were altered relative to the previously reported protocol. Extrudates exiting the die were cut into consistent lengths and dried in a forced-air oven at 80 °C for 10 min, then cooled to ambient temperature and sealed in aluminum-laminate pouches. Before instrumental testing, all prototype samples were conditioned at 25 °C and 50% relative humidity for 24 h to minimize moisture-related variability in fracturability and bolus rheology. This standardized conditioning ensured comparable moisture states across all samples, which is the primary determinant of glass -transition-related mechanical behavior in low-moisture extruded snack systems. Intact snacks were used for acoustic–mechanical and sensory analyses, while snack–saliva boluses for tribology were prepared by grinding extrudates to 50-mesh powder, hydrating them with artificial saliva, and allowing them to equilibrate under controlled conditions to simulate oral processing. All measurements were performed using snacks from the same production batch to ensure consistency across analytical platforms.

### 2.3. Acoustic–Mechanical Measurement

The mechanical and acoustic fracture behavior of extruded snack samples was evaluated using a texture analyzer (TA-XTplus^®^, Texture Technologies Corp., Scarsdale, NY, USA) equipped with a 50-kg load cell and controlled by Texture Exponent software (v6.2.6.0; Stable Micro Systems Ltd., Godalming, UK). An Acoustic Envelope Detector (AED; A/RAED) fitted with a ½ in pre-polarized condenser microphone was integrated for synchronous acquisition of force and airborne acoustic signals. Force values are reported in instrument gram-force units (g), as provided directly by the texture analyzer.

Two large-deformation test configurations were employed to probe complementary fracture mechanisms: bulk shearing using a five-blade Allo-Kramer (5AK) shear cell (Application Study REF: SNK1/KS5) and flexural snapping using a three-point bending (3PB) rig (Application Study REF: PTZ1/3PB). Each configuration was tested in ten independent replicates per sample.

#### 2.3.1. Mechanical Testing Configurations

For bulk shearing, approximately 9–10 g of sample was loaded into the Allo-Kramer shear cell to 50% capacity immediately prior to testing, following [16]. Samples were compressed at a crosshead speed of 2.00 mm/s to a distance of 45 mm. Force–distance curves typically exhibited multiple rupture peaks associated with progressive collapse of internal cell walls in expanded snack structures.

For three-point bending, individual snack sticks were positioned centrally on two lower supports mounted on a heavy-duty platform, with a fixed span selected according to sample geometry. A central loading blade applied downward displacement at 1.0 mm/s to a travel distance of 3 mm or until fracture. This configuration generated flexural snapping failure, enabling evaluation of bending resistance and brittleness. Samples of comparable dimensions were selected to minimize geometric variability.

#### 2.3.2. Acoustic Signal Acquisition

**Figure 2:**
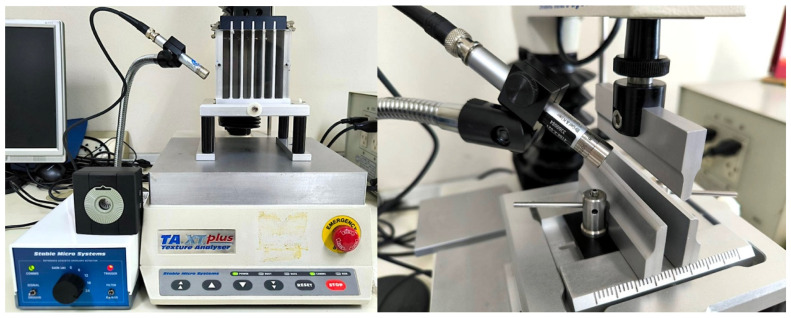
Instrumental setups for acoustic–mechanical testing: (**left**) bulk shearing with a five-blade Allo-Kramer shear cell and AED microphone positioned toward the fracture zone; (**right**) three-point bending configuration showing central loading blade, lower supports, and microphone alignment for capturing snapping sounds.

During both tests, airborne acoustic emissions were captured synchronously at 500 Hz using the AED system. The microphone was positioned at a 45° angle and approximately 1 cm from the fracture zone. Acquisition parameters were fixed across all tests (gain = 24 dB; envelope corner frequency = 3.125 kHz), in accordance with AED technical specifications (acoustic input range 1–12 kHz). AED output voltage and signal conditioning were managed automatically by the Exponent interface (Figure 2).

#### 2.3.3. Mechanical–Acoustic Signal Processing and Parameter Extraction

Force–acoustic signals were post-processed using a baseline-referenced workflow to quantify mechanical resistance, acoustic emission, and their coupling during fracture. Force onset (t = 0) was defined as the first time point at which force exceeded 5 g. The pre-onset segment (t < 0) was used to estimate baseline force and acoustic noise (mean ± SD).

Mechanical descriptors extracted from the force–time curve included maximum force and its occurrence time, compression work (force–time integral from onset to test end), and stiffness. Stiffness was defined as the mean positive loading rate (dF/dt) between force onset and the point at which force first reached 50% of maximum force, thereby representing initial structural rigidity while minimizing post-fracture oscillation effects.

Acoustic fracture events were detected using an adaptive threshold defined as baseline acoustic mean + 3 SD. Extracted acoustic descriptors included event count, timing of first and last events, acoustic activity duration, event density, maximum acoustic amplitude and its time, and acoustic energy. Acoustic energy was calculated as the time integral of the baseline-corrected acoustic signal, with negative deviations truncated at zero to retain only fracture-related emissions.

Coupled mechanical–acoustic descriptors included mean force at acoustic event times, the mechanical–acoustic ratio (acoustic energy normalized by mechanical work), and the absolute time difference between force and acoustic maxima. When no acoustic events were detected, event-based parameters were assigned a value of zero to retain all observations for multivariate analysis. All time-dependent parameters are reported relative to force onset. The complete parameter list and processing code are provided as Supplementary Material (Table S1 and Section S1.3 in Supplementary File S1).

#### 2.3.4. Multivariate and Index-Based Analysis of Crispness

Principal Component Analysis (PCA) was used to explore relationships among mechanical–acoustic parameters and to visualize differences among samples and testing configurations. Prior to PCA, variables were standardized using z-score normalization. Variable screening combined statistical and mechanistic criteria: variables with near-zero variance or unstable behavior were removed; strongly correlated variables (|*r*| > 0.80) were reduced to a single representative; and variables with low contribution (<10%) and poor representation (cos^2^ < 0.20) on the first two principal components were excluded.

Based on this screening, five variables were retained: maximum force, stiffness, time to maximum force, time to maximum acoustic emission, and acoustic energy. These parameters collectively represent mechanical resistance, fracture kinetics, and acoustic emission intensity. PCA was performed on the correlation matrix using XLSTAT (v2025.1.2, Addinsoft, Paris, France). The first two components were retained for interpretation based on eigenvalues, scree plots, and cumulative explained variance (>70%). Testing attachment (3PB or 5AK) was included as a qualitative supplementary variable.

Radar Plot Visualization: For radar plot visualization of crispness-driving features, the five PCA-screened variables were normalized by z-score standardization across the merged dataset. Mean normalized values were plotted for each sample and testing configuration to enable comparison of crispness profiles between bending- and shear-dominated fracture modes.

Crispness Index (CI): A composite Crispness Index (CI) was calculated to integrate mechanical resistance and acoustic emission into a single descriptor. The five PCA-screened variables were normalized using min–max scaling to a [0–1] range and averaged with equal weighting. CI values were computed separately for 5AK and 3PB datasets. Differences among samples were evaluated using one-way ANOVA with sample as a fixed factor, reporting least-squares means and Tukey’s HSD post hoc tests (*p* < 0.05). Residual normality was verified using the Shapiro–Wilk test.

### 2.4. Tribometry

#### 2.4.1. Oral Bolus Preparation

To simulate the chewed bolus phase of oral processing, each snack sample was ground using a laboratory mill and passed through a 60-mesh sieve. Two grams of the powdered sample were mixed with 0.5 mL of artificial saliva, gently stirred, and equilibrated at 37 °C for 1 min. The resulting hydrated paste was intended to mimic the oral bolus before swallowing and was used directly for tribological testing.

#### 2.4.2. Oral Tribology Measurements

Tribological measurements were performed using a ball-on-three-pins tribo-rheometry setup (MCR 302 Rheometer, Anton Paar GmbH, Graz, Austria), following the method described by [16], with modifications based on [24]. Approximately 3 g of the prepared bolus was carefully loaded into the tribology cell and spread to fully cover the three stationary polydimethylsiloxane (PDMS) pins.

Measurements were conducted at 37 °C under a constant normal force of 1 N. A 0.5 in (12.7 mm) soda-lime glass ball was mounted on the rotating shaft and brought into contact with the PDMS pins. Prior to each measurement, 0.3 mL of artificial saliva was added to the tribology cell and pre-sheared at 40 mm/s for 15 s to simulate initial oral lubrication. Then, 0.3 mL of the bolus sample was introduced, and the entrainment speed was logarithmically swept from 0.1 to 1000 mm/s. Friction coefficients (*μ*) were continuously recorded using RheoCompass™ software (Version 1.30, Anton Paar GmbH).

**Figure 3:**
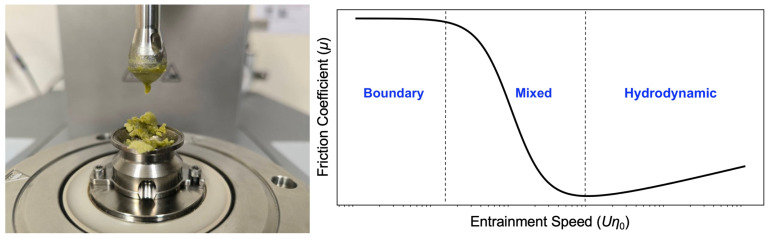
(**left**) Tribology configuration showing glass ball and bolus-loaded PDMS pins; (**right**) Schematic illustration of a Stribeck curve depicting boundary, mixed, and hydrodynamic lubrication regimes; the diagram is conceptual and not drawn to scale.

Stribeck curves were generated by plotting the friction coefficient (*μ*) against the entrainment parameter (*Uη*_0_), where *U* is the sliding speed (mm/s) and *η*_0_ is the zero-shear viscosity of the bolus (Pa·s), estimated from flow curve data at low shear rate. The resulting curves were used to evaluate the boundary, mixed, and hydrodynamic lubrication regimes for each sample (Figure 3).

#### 2.4.3. Determination of Lubrication Regime Transitions

To extract lubrication regime transition points, Stribeck curves were analyzed using a locally weighted scatterplot smoothing (LOWESS) procedure with a span of 0.2 to reduce experimental noise while preserving the curve’s structural features. All data were analyzed in log–log coordinates to capture nonlinear behavior across regimes.

Lubrication regime transitions were identified using a combination of derivative- and feature-based detection. The boundary-to-mixed transition (*Uη*_0__boundary → mixed) was defined as the first point where the slope of log(*μ*) versus log(*Uη*_0_) became strongly negative, exceeding a predefined threshold (slope < −0.05), reflecting the onset of shear-induced film formation. The mixed-to-hydrodynamic transition (*Uη*_0__mixed → hydrodynamic) was identified as the global minimum of the smoothed curve, indicating the point at which full fluid-film lubrication was established and friction began to increase again due to viscous drag. Each transition point was extracted independently for each sample.

Entrainment speed (*U*) was recorded in mm/s as provided by the instrument; therefore, the calculated entrainment parameter (*Uη*_0_) is expressed in units of Pa·mm throughout the manuscript.

The LOWESS and regime transition detection routine is provided in Supplementary File S1 (Section S1.4).

### 2.5. Sensory Evaluation

#### 2.5.1. Rationale and Attribute Selection

Texture perception of crispy extruded snacks arises from a sequence of oral-processing events, including initial bite resistance, fracture and sound emission, and subsequent bolus breakdown and lubrication. To capture these distinct perceptual stages while maintaining parsimony, four texture attributes—Hardness, Crunchiness, Sound Intensity, and Grittiness/Coarseness—were selected for sensory evaluation.

Attribute selection was guided by three complementary considerations: (i) prior sensory literature on extruded snack texture [9], (ii) relevance to different stages of oral processing, and (iii) direct alignment with instrumental mechanical–acoustic and oral tribological measurements obtained in this study. Hardness reflects force resistance during initial bite compression; Crunchiness captures the temporal pattern and abruptness of fracture events; Sound Intensity represents the loudness of fracture-associated acoustic emission at first bite; and Grittiness/Coarseness describes late-stage particle perception and lubrication-related mouthfeel during chewing. In this study, Crunchiness is used as the primary sensory descriptor corresponding to the perceptual construct commonly referred to as crispness in extruded snacks, as it captures the sharpness, distinctness, and temporal pattern of fracture events during biting, while Sound Intensity separately represents fracture loudness.

Operational definitions of the four attributes, together with their corresponding instrumental parameters, are summarized in Table 2. Attribute development followed ISO guidelines for sensory descriptor generation (ISO 13299:2016) [25] and builds on the descriptor framework previously established for extruded okara-based snacks [16]. This mechanistic alignment ensured that each attribute represented a distinct physical aspect of texture perception, minimizing redundancy and facilitating interpretation of sensory–instrumental relationships.

#### 2.5.2. Panel Characteristics

Twelve semi-trained panelists were recruited from the volunteer pool of the Sensory and Consumer Research Unit at the Institute of Food Research and Product Development (IFRPD), Kasetsart University, Bangkok, Thailand. Panelists were classified as semi-trained, defined as individuals with prior participation in descriptive and discriminative sensory studies but without extensive formal training hours [25,26]. All panelists had previous experience evaluating extruded snack products and were familiar with okara-based formulations through earlier studies [16].

All participants were 18 years of age or older and provided informed consent prior to participation. The sensory evaluation protocol was conducted in accordance with international guidelines for research involving human subjects and was approved by the Kasetsart University Research Ethics Committee (COE No. COE65/026). The panel size was consistent with recommendations for trained or semi-trained panels used in focused texture evaluation and discrimination studies with a limited and well-defined attribute set [26,27].

#### 2.5.3. Sensory Intensity Rating

Sensory intensity evaluation was conducted following the approach of [9], with adaptations to the selected attributes and product set. Panelists evaluated the perceived intensity of the four texture attributes—Hardness, Crunchiness, Sound Intensity, and Grittiness/Coarseness—across six snack samples comprising three okara-based prototypes (0%, 29%, and 40% okara) and three commercial benchmarks (NuMunchees, SnackJack, and Cheetos).

Each attribute was rated using a 100-mm unstructured line scale anchored at “low” (0 mm) and “high” (100 mm), consistent with standard sensory profiling practice [27]. Samples (3–5 pieces) were presented monadically in randomized order using three-digit blinding codes generated by a balanced Latin-square design to minimize order and carryover effects [25]. Evaluations were conducted in individual sensory booths under white lighting at 22 ± 2 °C, with water provided for palate cleansing between samples. All samples were evaluated within a single session.

Data processing and statistical analysis: Intensity ratings were recorded as line-scale distances (mm). Sample effects were assessed using one-way analysis of variance (ANOVA), followed by Tukey’s honestly significant difference (HSD) test at a significance level of α = 0.05. Multivariate relationships among samples and attributes were explored using principal component analysis (PCA) based on the correlation matrix, as recommended for sensory profiling data [26].

#### 2.5.4. Sensory Ranking and Multivariate Integration

Following intensity evaluation, panelists independently ranked the six snack samples for each attribute, with rank 1 indicating the lowest perceived intensity and rank 6 the highest. This ranking task was designed according to Flash Profile-type principles to enhance discrimination among samples while reducing scaling bias and inter-individual differences in scale use [4,28].

Data screening and GPA: Ranking data were screened using attribute-wise Spearman rank correlation against the panel consensus. One panelist exhibiting systematic inverse ranking behavior was corrected by rank reversal (*n* + 1 − rank), while another panelist showing unstable rankings across attributes was excluded from further analysis. The remaining panelists constituted the final dataset for multivariate analysis.

Generalized Procrustes Analysis (GPA) was applied separately to each attribute using the Commandeur method to account for individual differences in configuration and scale use [29,30,31]. Consensus significance was evaluated using permutation testing (500 permutations). For all attributes, the first consensus dimension (F1), explaining 100% of the variance, was retained as the representative sensory coordinate.

Integrated sensory mapping: The F1 consensus coordinates for all four attributes were assembled into a samples × attributes matrix. Principal component analysis (correlation scaling) was then applied to generate a global sensory map summarizing multivariate relationships among samples and texture attributes. This integrated sensory representation enabled direct qualitative and quantitative comparison with instrumental mechanical–acoustic and oral tribological measurements reported in subsequent sections.

### 2.6. Data Processing and Statistical Analysis

All data processing and statistical analyses were performed in XLSTAT (v2025.1.2, Addinsoft, Paris, France) unless otherwise stated. Sample sizes were as follows: proximate composition analyses were conducted in triplicate (*n* = 3); mechanical–acoustic measurements were performed with at least five independent replicates per sample (*n* ≥ 5); oral tribology measurements were conducted in triplicate (*n* = 3); and sensory evaluations were performed by twelve panelists (*n* = 12), as detailed in the corresponding Section 2.

For univariate comparisons, one-way ANOVA was applied with sample as a fixed factor, followed by Tukey’s HSD (α = 0.05). Model assumptions were checked using residual diagnostics and the Shapiro–Wilk test for normality. For multivariate analyses, PCA was conducted on the correlation matrix after appropriate scaling (z-score standardization for PCA; min–max scaling for index construction where specified). The suitability of the dataset for PCA was verified using the Kaiser–Meyer–Olkin (KMO) measure of sampling adequacy and Bartlett’s test of sphericity. The KMO value exceeded the recommended threshold of 0.60, and Bartlett’s test was significant (*p* < 0.001), confirming that the correlation structure was appropriate for principal component analysis.

For oral tribology, lubrication regime transition points (boundary → mixed and mixed → hydrodynamic) were identified from changes in slope and curvature of the friction coefficient–entrainment parameter (*Uη*_0_) relationships rather than from discrete phase boundaries. Accordingly, partial overlap in reported transition values among samples was considered inherent to the continuous nature of Stribeck behavior.

To examine the association between instrumental crispness and sensory perception, Spearman rank correlation analysis was performed between the Crispness Index (CI) and sensory Crunchiness using formulation-level least-squares mean values. Spearman correlation was selected due to the ordinal nature of formulation groups and the absence of replicate-level pairing between instrumental and sensory datasets.

For ranking data, panel consistency was screened using Spearman correlation against consensus, and GPA consensus significance was evaluated using permutation testing (500 permutations).

## 3. Results

### 3.1. Mechanical and Acoustic Characteristics of Extruded Snacks

**Figure 4:**
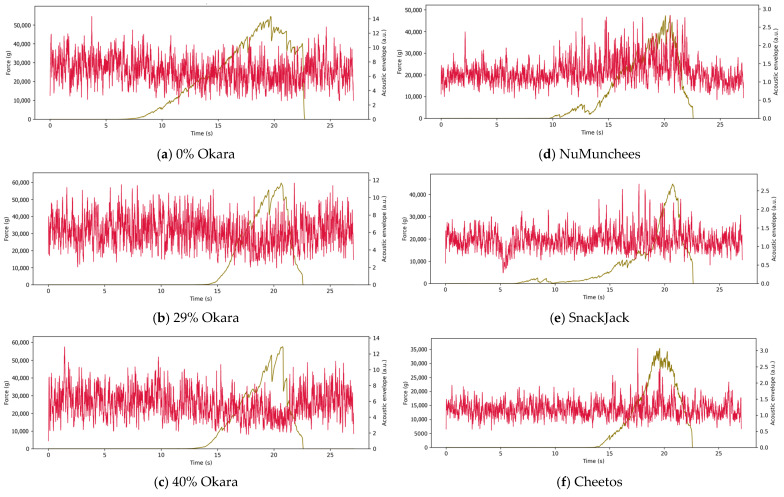
Representative force–time curves (**brown lines**, left axis) and synchronized SNR-enhanced acoustic envelope signals (**red lines**, right axis) obtained using the five-blade Allo–Kramer (5AK) shear cell. Panels show (**a**) 0% okara, (**b**) 29% okara, (**c**) 40% okara, and commercial benchmarks (**d**) NuMunchees, (**e**) SnackJack, and (**f**) Cheetos. Force–time profiles (left axis) reflect bulk fracture resistance, while acoustic envelopes (right axis, arbitrary units) capture sound emission associated with fracture events during shear.

**Figure 5:**
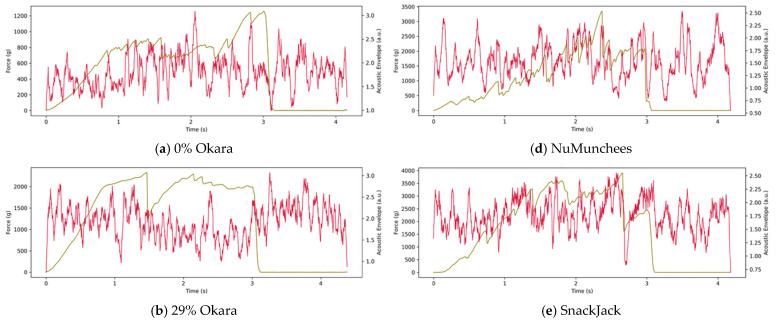

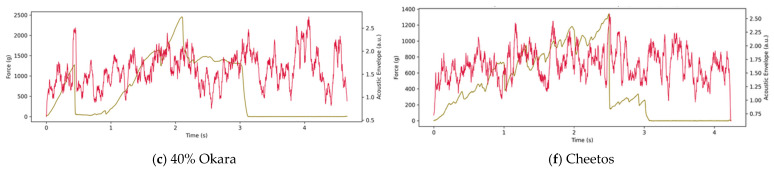
Representative force–time curves (**brown lines**, left axis) and synchronized SNR-enhanced acoustic envelope signals (**red lines**, right axis) obtained from three-point bending (3PB) tests. Panels show (**a**) 0% okara, (**b**) 29% okara, (**c**) 40% okara, and commercial benchmarks (**d**) NuMunchees, (**e**) SnackJack, and (**f**) Cheetos. Force–time profiles (left axis) reflect flexural fracture resistance, and acoustic envelopes (right axis, arbitrary units) capture sound emission associated with crack initiation and propagation during bending.

Representative force–time curves synchronized with SNR-enhanced acoustic envelope signals are shown in Figure 4 (5AK shear) and Figure 5 (3PB bending). In both configurations, force–time profiles are presented together with acoustic envelopes derived from the raw acoustic channel to visualize fracture-associated sound emission and facilitate comparison among formulations.

In the 5AK test (Figure 4), commercial snacks (NuMunchees, SnackJack, and Cheetos) exhibited rapid force increases to peak values of approximately 40,000–60,000 g, followed by abrupt force drops occurring within <1–2 s. These catastrophic failures coincided with intense, narrowly distributed acoustic envelope peaks, indicating dense fracture activity during bulk shear. In contrast, okara-based prototypes (0%, 29%, and 40%) showed smoother force–time profiles with comparable or slightly higher peak forces but more gradual force decay over several seconds. Their acoustic envelopes were markedly attenuated and temporally broadened, reflecting reduced fracture intensity.

Increasing okara content progressively modified the force–acoustic response. The 0% okara control displayed intermediate behavior, while the 29% formulation exhibited increased force resistance and further suppression of acoustic amplitude. The 40% okara sample showed the most gradual force evolution and weakest acoustic response across the deformation cycle.

Similar trends were observed under flexural loading in the 3PB test (Figure 5). Commercial snacks fractured rapidly, producing sharp force drops aligned with intense acoustic bursts indicative of rapid crack initiation and propagation. Okara-based samples exhibited delayed fracture, smoother force–time evolution, and substantially lower acoustic envelope intensities. Again, the 40% okara formulation showed the longest deformation time prior to fracture and the lowest acoustic activity, consistent with increased resistance to crack initiation and propagation.

Across both deformation modes, the representative curves consistently distinguished brittle fracture behavior—characterized by short fracture times, abrupt force loss, and concentrated acoustic activity—from tougher, damped fracture behavior associated with okara incorporation, where force dissipation occurred over longer timescales with reduced sound emission.

### 3.2. Multivariate Discrimination of Crispness Attributes

**Figure 6:**
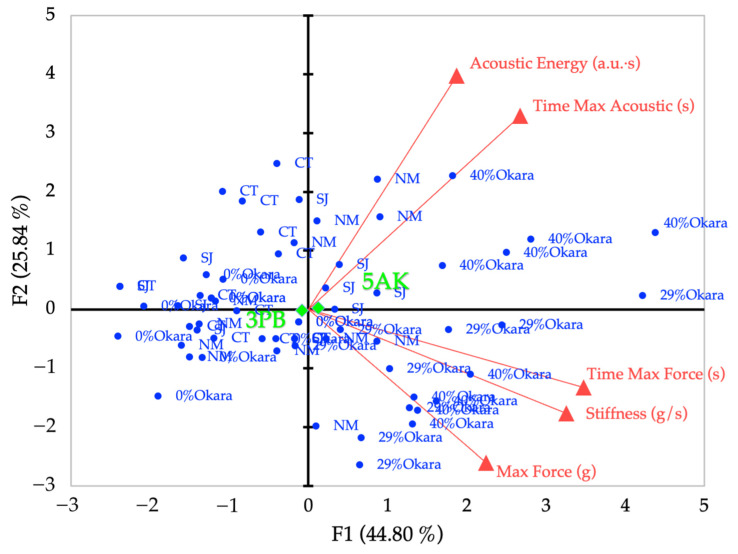
Principal component analysis (PCA) biplot of screened mechanical–acoustic crispness variables derived from combined three-point bending (3PB) and five-blade Allo–Kramer (5AK) shear tests. Blue points represent individual observations for okara-based prototypes (0%, 29%, and 40% okara) and commercial benchmark snacks (NuMunchees, SnackJack, and Cheetos). Red vectors represent active variables: maximum force, stiffness, time to maximum force, acoustic energy, and time to maximum acoustic event. Green diamonds indicate the centroids of the testing attachments (3PB and 5AK), included as supplementary qualitative variables. The first two principal components account for 70.64% of the total variance (F1 = 44.80%, F2 = 25.84%).

PCA of the five screened mechanical–acoustic variables yielded a clear and interpretable structure, with the first two principal components (F1 and F2) explaining 70.64% of the total variance (F1 = 44.80%, F2 = 25.84%; Figure 6). Eigenvalues of subsequent components were below unity, indicating that the main variance structure was adequately captured in two dimensions.

Loadings showed that F1 was primarily associated with mechanical fracture resistance, with strong positive correlations for time to maximum force (r = 0.84), stiffness (r = 0.79), and maximum force (r = 0.54). In contrast, F2 was dominated by acoustic-related variables, particularly acoustic energy (r = 0.73) and time to maximum acoustic event (r = 0.60), indicating partial decoupling of force-driven fracture behavior and acoustic emission characteristics.

In the biplot, snack samples were distributed along F1 according to mechanical resistance and fracture severity, while separation along F2 reflected differences in the timing and intensity of acoustic release. The supplementary qualitative variable “attachment type” projected near the origin, indicating that attachment configuration did not define the principal axes but modulated sample positioning. Centroids of 3PB and 5AK measurements showed minor shifts mainly along F1, suggesting differences in mechanical sensitivity rather than fundamentally distinct fracture mechanisms.

### 3.3. Crispness-Driving Variables

**Figure 7:**
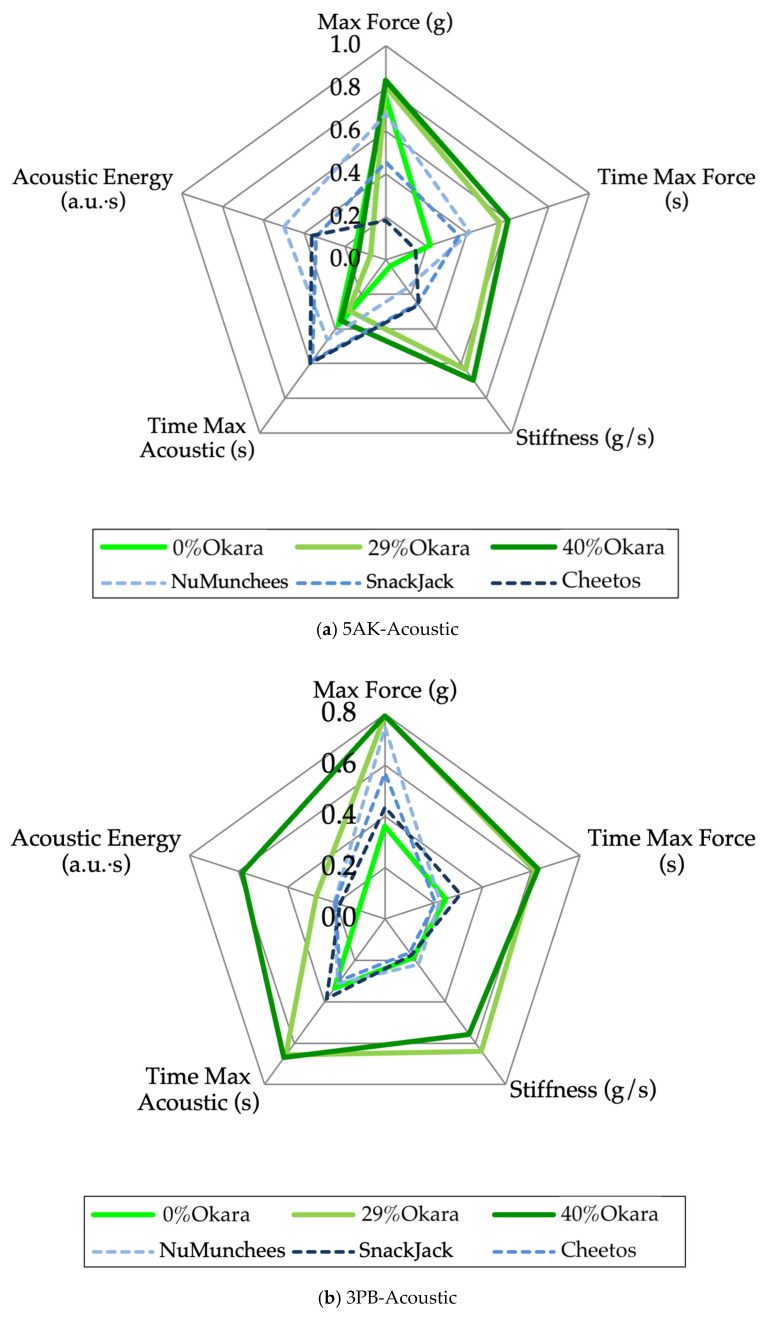
Radar plots of normalized crispness-driving mechanical–acoustic variables for extruded snack samples: (**a**) five-blade Allo-Kramer shear with acoustic detection (5AK-acoustic) and (**b**) three-point bending with acoustic detection (3PB-acoustic). Variables include maximum force, time to maximum force, stiffness, time to maximum acoustic event, and acoustic energy, min–max normalized (0–1) across all samples. Solid green lines represent okara-based prototypes (0%, 29%, 40%), and dashed blue lines represent commercial benchmarks (NuMunchees, SnackJack, Cheetos).

Radar plots of the screened crispness-driving variables revealed clear and systematic differences between okara-based prototypes and commercial benchmarks, as well as between testing attachments (Figure 7). In the 5AK-acoustic configuration (Figure 7a), the 40% okara sample exhibited the highest normalized values for maximum force, stiffness, and time to maximum force, indicating a mechanically stronger structure requiring greater resistance to fracture. These features were accompanied by relatively low acoustic energy and shorter time to maximum acoustic response, consistent with limited crack propagation and attenuated sound emission during bulk shearing.

The 0% okara control showed lower force-related parameters but moderately elevated acoustic responses, reflecting a more brittle, starch-dominated matrix. The 29% okara formulation exhibited intermediate behavior, bridging the mechanical–acoustic profiles of the control and high-fiber prototype.

Commercial benchmark snacks displayed distinct profiles characterized by higher acoustic energy and delayed acoustic maxima relative to force parameters, consistent with highly expanded, porous structures that favor audible fracture. SnackJack showed a more balanced profile, positioning it closer to the lower-okara formulations.

A similar but not identical pattern was observed in the 3PB-acoustic test (Figure 7b). While force-related parameters remained elevated for the 40% okara sample, acoustic differences among samples were more pronounced than in the 5AK test, indicating greater sensitivity of bending-induced fracture to surface and cell-wall heterogeneity. Overall, the radar plots demonstrate that attachment type influences the relative weighting of mechanical versus acoustic attributes while preserving consistent trends associated with okara inclusion.

### 3.4. Crispness Index and Practical Implications

**Figure 8:**
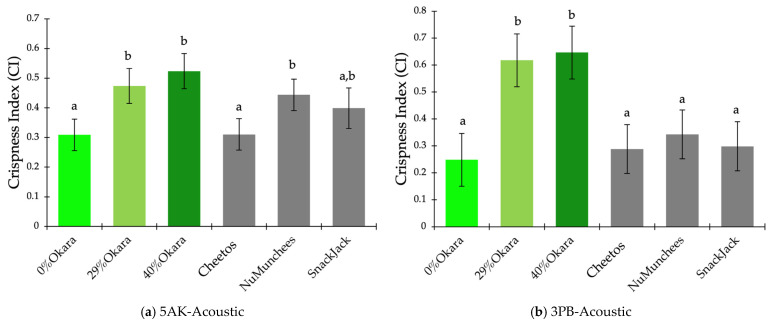
Crispness Index (CI) of extruded snack samples calculated from PCA-screened, normalized mechanical–acoustic variables using (**a**) 5AK bulk shearing–acoustic tests and (**b**) 3PB–acoustic tests. Bars represent least-squares means ± SE. Green bars indicate okara-based prototypes (0%, 29%, and 40%), and grey bars represent commercial benchmark snacks (Cheetos, NuMunchees, and SnackJack). Different lowercase letters indicate significant differences among samples within each panel (one-way ANOVA, Tukey’s HSD, *p* < 0.05).

For the 5AK bulk shearing–acoustic test, CI values differed significantly among samples (*p* < 0.0001; Figure 8a). The 40% okara snack exhibited the highest CI, followed by the 29% okara snack and NuMunchees, whereas the 0% okara and Cheetos samples showed the lowest CI values and were not significantly different. SnackJack occupied an intermediate position. LS-mean CI values ranged from approximately 0.31 to 0.52, indicating moderate but systematic separation driven by combined mechanical resistance and acoustic output.

In contrast, the 3PB–acoustic test produced a wider CI range and stronger discrimination among formulation groups (*p* < 0.0001; Figure 8b). Both 29% and 40% okara snacks formed a distinct high-CI group (CI > 0.60), while commercial benchmarks and the 0% okara control clustered in a significantly lower CI group. Residual diagnostics confirmed normality for both ANOVA models (Shapiro–Wilk, *p* > 0.05).

Spearman’s rank correlation analysis performed using formulation-level LS-mean values revealed a moderate inverse association between the Crispness Index (CI) and sensory Crunchiness for both mechanical–acoustic test configurations (shear- and bending-based tests: ρ = −0.60, *p* = 0.242). The identical correlation coefficients reflect the highly consistent rank ordering of formulations produced by the two test configurations, rather than replicate-level pairing between instrumental and sensory datasets. Given the limited number of formulation-level observations and the independent acquisition of instrumental and sensory data, this association is interpreted as indicative of directional concordance rather than a predictive relationship.

### 3.5. Oral Tribological Behavior

**Figure 9:**
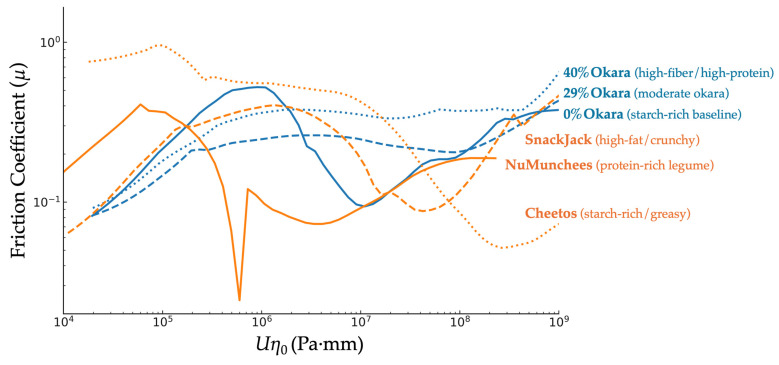
Experimental Stribeck curves showing friction coefficient (*μ*) as a function of the entrainment parameter (*Uη*_0_, Pa·mm) for okara-based extruded snacks (blue) and commercial benchmarks (orange). Numerical axis values represent experimentally measured data (not schematic scaling). Lubrication regimes (boundary, mixed, hydrodynamic) are shown conceptually in Figure 3.

Stribeck curves (Figure 9) revealed pronounced differences in lubrication behavior between okara-based prototypes and commercial benchmarks. While all samples exhibited boundary, mixed, and hydrodynamic regimes, okara-based snacks showed a systematically prolonged boundary regime, transitioning into mixed lubrication at higher entrainment parameters (*Uη*_0_ ≈ 1.0 × 10^5^–3.5 × 10^5^) compared with the commercial benchmarks (*Uη*_0_ ≈ 7.0 × 10^4^–2.0 × 10^5^; Table 3). This shift indicates delayed lubricant film formation and more persistent asperity contact in the fiber-rich okara systems.

Because lubrication regime boundaries were identified from changes in the slope of friction–*Uη*_0_ relationships rather than from discrete phase transitions, partial overlap in transition values between samples is expected.

In contrast, benchmark snacks—particularly the legume-based NuMunchees and the high-fat SnackJack—entered the mixed lubrication regime at lower *Uη*_0_ values, reflecting earlier bolus spreading and more effective surface lubrication. The transition from mixed to hydrodynamic lubrication occurred at substantially higher *Uη*_0_ values for okara-based snacks (7.5 × 10^7^–1.6 × 10^8^) than for most benchmarks, with the exception of Cheetos, which exhibited a delayed hydrodynamic transition due to its starch-rich yet lipid-dominated bolus structure.

Friction decreased rapidly with increasing entrainment speed for commercial snacks, reflecting efficient lubrication. Okara prototypes showed a slower frictional decline, consistent with cohesive, fiber-rich boli that limited spreading and delayed film formation. At 40 mm/s, okara-based snacks exhibited high friction coefficients (0.26–0.27), whereas benchmarks showed significantly lower values (0.085–0.097). At 100 mm/s, all samples entered the hydrodynamic regime, with okara snacks showing the highest friction (0.28–0.31) and Cheetos showed the lowest (0.058), reflecting differences in bolus viscosity and lipid release.

**Figure 10:**
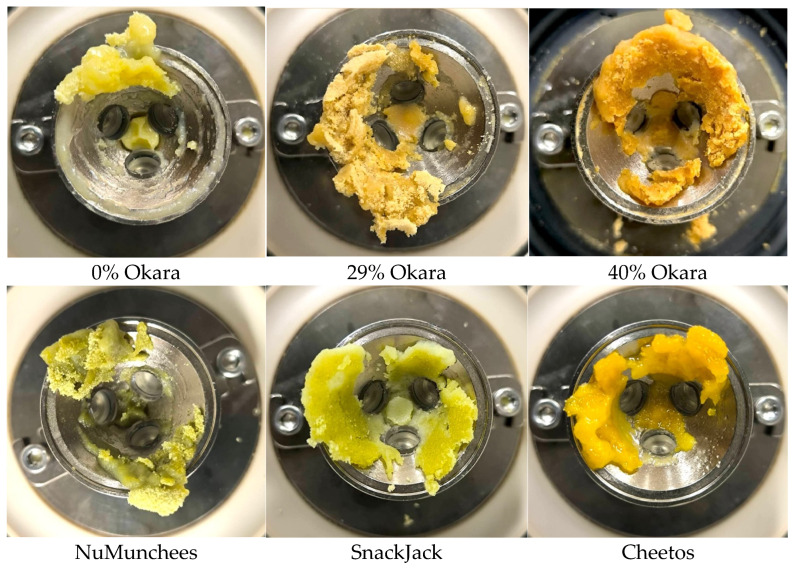
Representative images of bolus-like residues remaining on the PDMS–glass tribometer surfaces after oral tribology testing. Okara-based prototypes (0%, 29%, 40%) and commercial benchmarks (NuMunchees, SnackJack, Cheetos) are shown.

Post-test residue images (Figure 10) supported these trends. Okara-based samples formed dense, cohesive residues with limited spreading, while benchmark snacks produced softer, more lubricious residues, particularly for the high-fat Cheetos sample.

### 3.6. Sensory Evaluation: Results and Discussion

#### 3.6.1. Sensory Intensity Ratings

**Figure 11:**
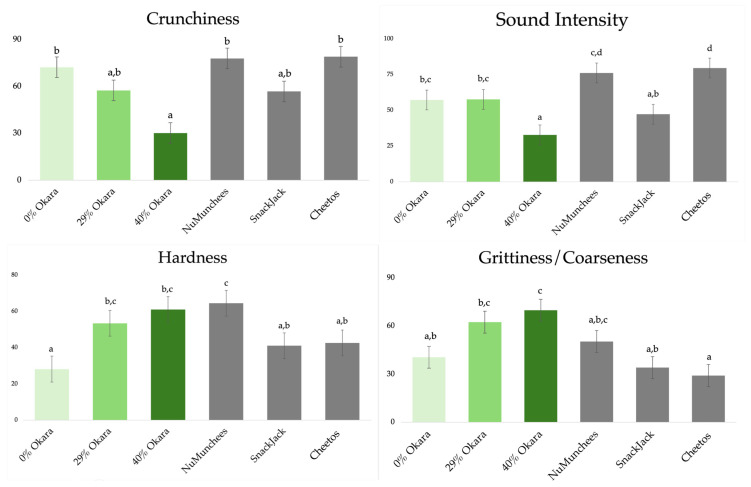
Sensory intensity ratings (least-squares means ± SE) for Crunchiness, Sound Intensity, Hardness, and Grittiness/Coarseness attributes across six snack samples. Green bars indicate okara-based prototypes (0%, 29%, and 40%), and grey bars represent commercial benchmark snacks (Cheetos, NuMunchees, and SnackJack). Different superscript letters indicate significant differences among samples within each attribute (Tukey’s HSD, *p* < 0.05).

Significant differences were observed among samples for all four sensory attributes (Crunchiness, Sound Intensity, Hardness, and Grittiness/Coarseness; *p* < 0.05). Cheetos and NuMunchees received the highest ratings for Crunchiness and Sound Intensity, whereas increasing okara content produced a clear dose-dependent reduction in these attributes and a concomitant increase in Hardness and Grittiness/Coarseness perception (Figure 11). The 40% okara sample was consistently rated as the hardest and Coarsenessst, while the 0% okara prototype and SnackJack exhibited intermediate profiles.

#### 3.6.2. Multivariate Analysis of Sensory Intensity Ratings

**Figure 12:**
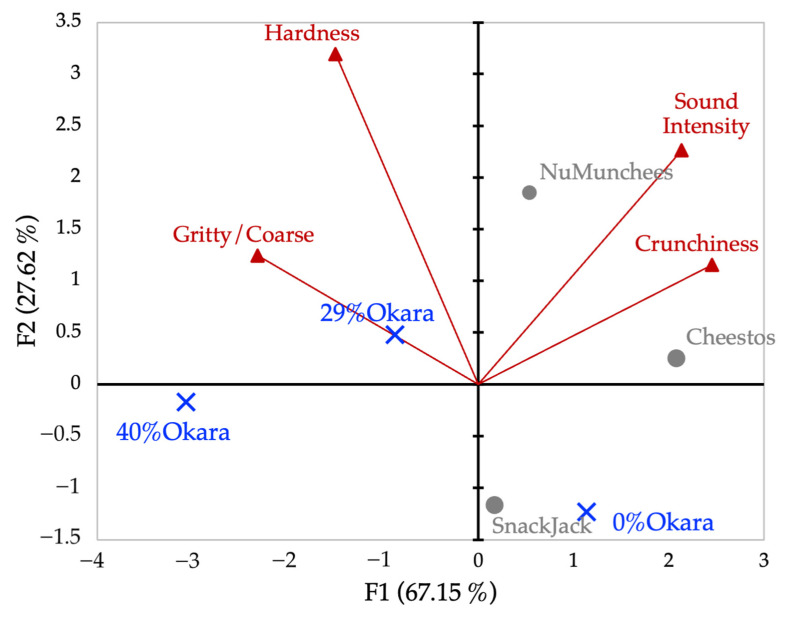
PCA biplot of sensory intensity ratings for four texture attributes. Red vectors represent attribute loadings (Crunchiness, Sound Intensity, Hardness, Grittiness/Coarseness), and points represent snack samples. The first two components account for 94.77% of the total variance (F1 = 67.15%, F2 = 27.62%).

PCA of sensory intensity ratings explained 94.8% of total variance on the first two components (F1 = 67.2%, F2 = 27.6%; Figure 12). F1 represented a dominant crisp–acoustic versus dense–fibrous axis, separating commercial snacks from high-okara prototypes. The 0% okara sample and SnackJack occupied intermediate positions.

#### 3.6.3. Sensory Ranking (Flash Profile) and GPA-Based Mapping

**Figure 13:**
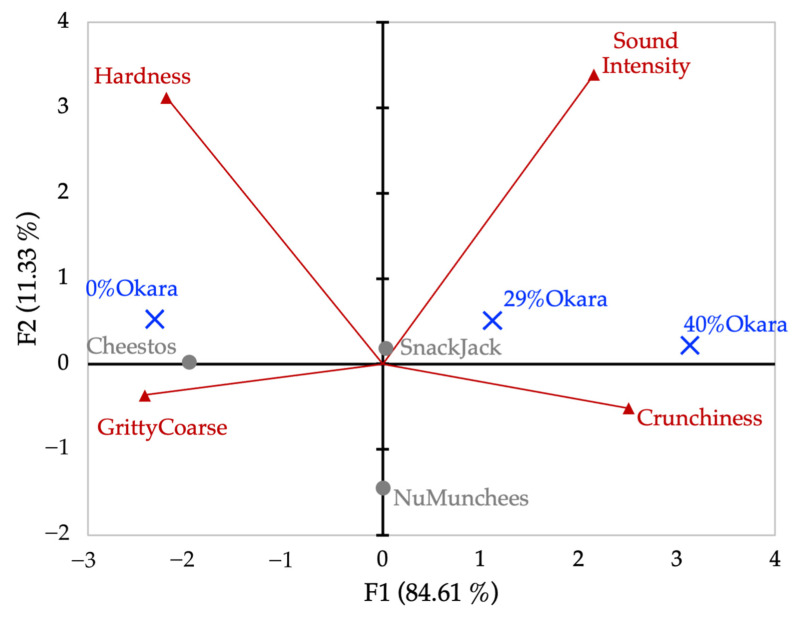
PCA biplot derived from Generalized Procrustes Analysis (GPA) consensus scores obtained from sensory ranking (Flash Profile) data. Blue symbols represent snack samples and red vectors represent sensory attributes. PCA was performed using correlation scaling. The first two components account for 95.94% of the total variance (F1 = 84.61%, F2 = 11.33%).

PCA of GPA consensus scores from ranking data explained 95.9% of total variance (Figure 13). The first component (F1 = 84.6%) mirrored the intensity-based sensory structure but provided stronger separation among okara formulations, particularly distinguishing 29% and 40% okara samples from the control and commercial benchmarks. Secondary variation along F2 differentiated commercial products.

## 4. Discussion

### 4.1. Mechanical–Acoustic Signatures of Fracture Behavior in Extruded Snacks

The combined force–acoustic measurements demonstrate that snack formulation strongly governs fracture mechanics and sound emission. Commercial benchmarks exhibited abrupt force drops accompanied by intense acoustic bursts, characteristic of highly expanded, thin-walled cellular structures undergoing brittle, catastrophic failure. Such fracture behavior is closely associated with high perceived crispness, as reflected in elevated sensory Crunchiness and Sound Intensity ratings, where rapid cell-wall rupture generates dense acoustic transients during deformation. Previous studies consistently report strong relationships between acoustic event density, acoustic amplitude, and sensory crispness-related attributes—such as Crunchiness and fracture sound—in expanded snacks [19,32], and recent work further confirms that acoustic descriptors can robustly predict crispness in puffed grain-based foods [6] and can track crispness loss during secondary shelf life of legume-based chips [7].

In contrast, okara-based snacks showed attenuated acoustic responses and smoother force–time profiles, indicating a transition toward tougher, less brittle fracture modes. The incorporation of okara—rich in insoluble dietary fiber and moisture-binding components—likely promotes thicker cell walls, reduced porosity, and lower expansion during extrusion. These formulation-driven microstructural changes restrict crack initiation and propagation, leading to gradual energy dissipation and suppressed fracture-related sound emission. Similar reductions in acoustic energy and fracture intensity have been reported for dense or fiber-enriched extrudates with lower expansion ratios [33,34], and recent studies on pulse- and lentil-enriched extruded snacks also demonstrate reduced expansion, higher density, and modified crispness attributes with increasing fiber or protein content [14,15,35].

The consistency of these trends across both five-blade Allo-Kramer shear and three-point bending tests confirms that okara-induced damping of fracture behavior is robust to deformation mode. Increasing okara content further accentuated this effect, with the 40% formulation exhibiting the highest deformation resistance and weakest acoustic activity. Together, these findings establish a mechanistic link between formulation-driven microstructural modification and the observed mechanical–acoustic response, providing the foundation for subsequent multivariate and index-based analyses.

### 4.2. Multidimensional Representation of Instrumental Crispness

The reduced PCA offers a physically meaningful and statistically robust representation of instrumental crispness. Restricting the analysis to five carefully screened variables increased the variance captured by the first two components, highlighting the importance of variable selection when integrating force and acoustic data. The resulting PCA structure revealed two complementary dimensions: a mechanical fracture axis associated with resistance to deformation and force magnitude, and an acoustic emission axis reflecting sound generation during fracture.

This decoupling aligns with established understanding of crispness perception as a multisensory attribute integrating tactile resistance and auditory feedback during mastication, consistent with sensory Crunchiness and Sound Intensity responses. Recent multisensory and structure–texture–acoustic analyses have reported similar decoupling in crispy breaded systems [10] and dynamic perception during oral processing [11]. Importantly, including test attachment as a supplementary variable demonstrated that both shear and bending measurements aligned along the same principal axes, indicating that the two configurations probe the same underlying fracture phenomena despite differences in stress distribution and sensitivity. This supports the use of a unified variable set for attachment-independent comparison of crispness behavior.

Beyond visualization, the PCA outcomes provide a rational basis for downstream analyses. The selected variables capture the dominant contributors to instrumental crispness and can be readily translated into radar plots and composite indices, in line with recent developments in crispness indices that combine acoustic and mechanical features into robust multivariate metrics [36]. This multiscale analytical strategy strengthens the link between instrumental fracture behavior and formulation-driven texture differences relevant to product development.

### 4.3. Crispness-Driving Variables and Composite Crispness Index

Radar-based visualization complements PCA by translating multivariate relationships into sample-specific fracture “fingerprints.” Expansion of the radar polygon with increasing okara content—driven primarily by force- and stiffness-related variables—confirms that okara reinforces the snack matrix, increasing resistance to deformation and delaying fracture onset. This behavior is consistent with the role of insoluble dietary fiber in thickening cell walls, restricting bubble growth, and promoting denser internal structures during extrusion.

Conversely, reduced acoustic energy and delayed acoustic responses in high-okara samples indicate dampened crack propagation and sound emission, reflecting a shift from brittle to more progressive fracture behavior and corresponding reductions in sensory Crunchiness and Sound Intensity. Commercial benchmarks exhibited profiles dominated by acoustic attributes rather than mechanical resistance, consistent with high sensory Crunchiness, strong Sound Intensity, and consumer-perceived ‘light and crunchy’ textures of highly expanded starch-based snacks.

Differences between shear- and bending-based radar plots underscore the importance of fracture mode in crispness characterization. Bulk shearing emphasizes cumulative resistance and energy dissipation across multiple fracture sites, whereas bending accentuates localized crack initiation and propagation. The stronger acoustic discrimination observed under bending suggests that flexural tests are particularly sensitive to surface brittleness and structural heterogeneity, while shear-based tests better capture bulk reinforcement effects.

Integration of PCA-guided variable screening with radar visualization enables a robust workflow for crispness analysis. Extending this approach, the composite Crispness Index (CI) consolidates normalized mechanical–acoustic parameters into a single, interpretable descriptor. CI values differentiated samples consistently across test configurations, supporting the construct validity of the index. Recent studies similarly demonstrate that composite or ratio-based indices integrating mechanical and acoustic metrics correlate strongly with perceived crispness and consumer liking [10,19,36,37], reinforcing the relevance of multivariate crispness indices in capturing key fracture–acoustic properties of crispy snacks.

### 4.4. Oral Tribology and Sensory–Instrumental Integration

Oral tribology results revealed that okara incorporation increases resistance to lubrication, prolongs boundary contact, and elevates friction across lubrication regimes, whereas commercial snacks—particularly fat-rich extrudates—exhibited earlier film formation and lower friction coefficients. These differences reflect compositional effects on bolus formation and lubrication behavior and provide mechanistic insight into expected sensory perceptions of dryness, roughness, and mouthcoating.

Sensory results integrate coherently with the instrumental findings. Crispness perception emerges from the combined effects of fracture mechanics and acoustic emission rather than force-based resistance alone, and in this study is reflected primarily by sensory Crunchiness and Sound Intensity rather than Hardness alone, consistent with established crispness theory [9,38].

The observed decline in sensory Crunchiness and Sound Intensity with increasing okara content corresponded closely to reduced acoustic event density and attenuated fracture sound measured instrumentally. Acoustic emission parameters have been shown to correlate strongly with sensory crispness (r ≈ 0.80–0.90), whereas force-based metrics exhibit weaker or inconsistent relationships [38]. Similarly, fracture sound levels of approximately 65–75 dB SPL have been associated with higher perceived crispness, and attenuation of sound reduces crispness perception even at comparable bite forces [39]. These relationships are further supported by recent work demonstrating the predictive power of acoustic features in puffed-grain systems [6] and by studies showing crispness loss reflected in acoustic–mechanical signatures during storage [7].

Increased sensory Hardness with fiber enrichment reflects enhanced mechanical resistance and delayed fracture. Insoluble dietary fiber has been shown to increase compression force at fracture by approximately 1.5–3× relative to fiber-free controls, accompanied by reduced expansion and thicker cell walls [12,13]. Sensory Grittiness/Coarseness perception aligns with the tribological findings, as rough or particulate mouthfeel is associated with sustained high friction coefficients in the boundary and mixed regimes (µ ≈ 0.2–0.4), whereas smoother systems exhibit substantially lower friction (µ < 0.1) [40,41]. Recent reviews further highlight the central role of lubrication and friction-driven mechanisms in graininess, creaminess, and roughness perception during oral processing [21,22,42].

Multivariate sensory maps integrate these mechanisms into a coherent structure–perception relationship. The primary sensory axis captured a transition from brittle, highly aerated structures to dense, fiber-rich matrices dissipating energy through progressive deformation and reduced sound emission. Similar correspondence between sensory and instrumental PCA structures has been reported previously, supporting the use of multivariate sensory mapping to infer underlying structural mechanisms [26,30]. Recent structure–texture–sensory mapping studies in reformulated cereal and baked products provide further evidence of these correspondences [43].

### 4.5. Methodological Implications and Texture Phenotype Framework

Integrating mechanical–acoustic measurements, oral tribology, and multivariate sensory analysis provides a more comprehensive characterization of extruded snack texture than single-domain approaches. Force-based measurements alone fail to capture fracture dynamics and oral processing phenomena governing texture perception in brittle foods [34]. The proposed framework overcomes these limitations by embedding instrumental measurements within an oral-processing context, as highlighted in recent rheology–tribology reviews emphasizing multi-domain approaches for complex texture characterization [21] and in contemporary mouthfeel frameworks advocating oral-processing–based interpretation of sensory attributes [22].

This integrated approach enables improved discrimination among fiber-enriched formulations, which are often poorly differentiated using conventional texture parameters despite clear sensory differences [12,13]. By linking mechanical resistance, fracture acoustics, and lubrication behavior to dominant sensory perceptions, the framework enables instrumental definition of texture phenotypes that are directly interpretable in sensory terms (Table 4). These phenotypes translate complex datasets into actionable texture descriptors, supporting rational formulation and optimization consistent with texture-driven and material-oriented food design principles.

This phenotype-based framework is particularly relevant for nutritionally enhanced extruded snacks, where increases in dietary fiber or protein fundamentally alter fracture behavior, sound emission, and lubrication during mastication, consistent with the growing body of work on high-protein and high-fiber extruded snacks [14,15,18]. By capturing these coupled effects across multiple stages of oral processing, the proposed approach addresses a key limitation in current texture research and provides a practical tool for designing sustainable, fiber-rich snacks with controlled texture performance.

The glass transition temperature (T_g_) of the extruded snacks was not directly measured in this study; therefore, the potential contribution of physical aging effects cannot be fully excluded. However, because all samples were equilibrated under identical temperature and humidity conditions prior to testing, any aging-related effects are expected to be systematic and unlikely to alter the comparative trends observed among formulations. Future work integrating thermal analysis (e.g., DSC or DMA) would further strengthen mechanistic interpretation.

## 5. Conclusions

This study demonstrates that texture perception in extruded snacks arises from the combined contributions of fracture mechanics, acoustic emission, and lubrication behavior during oral processing, rather than from force resistance alone. By integrating synchronized mechanical–acoustic testing, oral tribology, and multivariate sensory analysis, this work establishes a mechanism-based framework for characterizing texture in fiber-enriched extruded snack systems.

Across the prototype formulations, increasing okara content from 0% to 29% and 40% produced systematic and quantifiable changes in fracture and sensory behavior and associated sensory responses. Relative to the 0% control, the 40% okara snack exhibited a 45–60% reduction in acoustic energy, a 30–40% decrease in acoustic event count, and approximately 25% increase in time to maximum force, indicating a shift from brittle, sound-emitting failure toward damped, progressive deformation with attenuated acoustic emission. Sensory responses corroborated these instrumental patterns: perceived Crunchiness and Sound Intensity, reflecting crispness-related perception, decreased significantly (*p* < 0.05) with increasing okara, whereas Hardness increased and Grittiness/Coarseness perception became more pronounced. Oral tribology further revealed that high-okara snacks formed more cohesive, viscous boli with higher friction coefficients in the mixed regime (approximately 0.15–0.20, compared with 0.08–0.10 in the control), explaining late-stage Coarseness or pasty mouthfeel.

By converging instrumental and sensory findings, this study introduces instrumentally defined texture phenotypes that translate mechanical–acoustic signatures and lubrication characteristics into sensory-interpretable categories. This integrated, oral-processing-oriented approach enhances discrimination among fiber-enriched formulations and provides a practical, oral-processing-oriented framework for material-driven formulation and optimization of nutritionally enhanced extruded snacks.

## Figures and Tables

**Table 1:** Proximate composition (mean ± SD, % *w*/*w*) and classification of snack samples used in this study.

Sample	Type Role/Representation	Protein (%)	Fat (%)	Carbohydrate (%)	Fiber (%)	Moisture + Ash (%)
0% Okara	Prototype—Baseline (starch-rich)	8.61 ± 0.09 ^b^	0.55 ± 0.01 ^a^	79.18 ± 0.21 ^e^	3.46 ± 0.03 ^a^	8.20 ± 0.08 ^d^
29% Okara	Prototype—Moderate okara	15.70 ± 0.16 ^c^	1.63 ± 0.02 ^b^	51.45 ± 0.49 ^d^	22.22 ± 0.22 ^e^	9.00 ± 0.09 ^e^
40% Okara	Prototype—High-fiber/high-protein	17.78 ± 0.18 ^e^	1.97 ± 0.02 ^c^	42.41 ± 0.58 ^a^	28.70 ± 0.29 ^f^	9.14 ± 0.09 ^e^
NuMunchees	Commercial—Protein-rich legume	20.00 ± 0.20 ^f^	10.00 ± 0.10 ^d^	50.60 ± 0.49 ^d^	13.40 ± 0.13 ^d^	6.00 ± 0.06 ^c^
SnackJack	Commercial—High-fat/crunchy	16.10 ± 0.16 ^d^	25.80 ± 0.26 ^e^	43.40 ± 0.57 ^b^	9.70 ± 0.10 ^c^	5.00 ± 0.05 ^b^
Cheetos	Commercial—Starch-rich/greasy	6.70 ± 0.07 ^a^	40.00 ± 0.40 ^f^	46.21 ± 0.56 ^c^	4.09 ± 0.06 ^b^	3.00 ± 0.04 ^a^

Values are expressed as mean ± SD (*n* = 3). Different superscript letters within the same column indicate significant differences among samples (*p* < 0.05).

**Table 2:** Instrumental texture parameters and corresponding sensory attributes used for sensory evaluation of extruded snacks.

Instrumental Domain/Parameter	Operational Definition	Related Sensory Attribute	Sensory Definition
**Mechanical (force)**			
Maximum force	Peak force recorded during compression, shear, or bending prior to major fracture	Hardness	Force required to bite and compress the product at first bite
Time to maximum force	Time elapsed from probe contact to maximum force	Hardness	Resistance perceived before the product fractures
Stiffness	Initial slope of the force–time curve prior to fracture	Hardness	Perceived firmness during initial biting
**Mechanical–fracture pattern**			
Fracture time/force-drop pattern	Duration and abruptness of force decrease associated with fracture events	Crunchiness	Perception of how sharply and distinctly the product fractures during biting
Number/density of fracture events	Distribution of force drops over time during deformation	Crunchiness	Sensation of multiple versus single fracture events during chewing
**Acoustic**			
Acoustic energy	Integrated acoustic signal amplitude during fracture	Sound Intensity	Loudness of the sound produced at first bite
Maximum acoustic amplitude	Peak acoustic envelope amplitude during fracture	Sound Intensity	Strength of the audible cracking sound
Time to maximum acoustic event	Time at which the strongest sound occurs relative to bite	Crunchiness/Sound Intensity	Timing of sound relative to the biting action
**Oral tribology**			
Boundary-to-mixed regime transition (*Uη*_0_)	Entrainment speed required for lubrication film formation	Grittiness/Coarseness	Perception of roughness or particulate sensation during chewing
Friction coefficient (mixed regime)	Friction measured at oral-relevant sliding speeds (e.g., 40 mm/s)	Grittiness/Coarseness	Degree of roughness, graininess, or lack of smoothness in the mouth
Residue spreading behavior	Visual and tribological observation of bolus spreading after shear	Grittiness/Coarseness	Persistence of Coarseness particles or paste-like residue after chewing

**Table 3:** Lubrication regime transition points (*Uη*_0_) and friction coefficients of snack samples evaluated at representative entrainment speeds (10, 40, and 100 mm/s).

Sample	*Uη*_0_ (Pa·mm)		Friction Coefficient (*μ*)		
	Boundary → Mixed	Mixed → Hydrodynamic	10 mm/s	40 mm/s	100 mm/s
0% Okara	1.0 × 10^5^	1.0 × 10^7^	0.171 ^ab^	0.260 ^b^	0.280 ^b^
29% Okara	3.5 × 10^5^	7.5 × 10^7^	0.248 ^b^	0.270 ^b^	0.310 ^b^
40% Okara	2.0 × 10^5^	1.6 × 10^8^	0.257 ^b^	0.270 ^b^	0.300 ^b^
NuMunchees	7.0 × 10^4^	6.0 × 10^5^	0.089 ^a^	0.097 ^a^	0.130 ^a^
SnackJack	2.0 × 10^5^	1.1 × 10^6^	0.114 ^a^	0.088 ^a^	0.140 ^a^
Cheetos	1.2 × 10^5^	2.6 × 10^8^	0.189 ^ab^	0.085 ^a^	0.058 ^a^

^a,b^ Different superscript letters within a column indicate significant differences (*p* < 0.05; *n* = 3).

**Table 4:** Instrumentally defined texture phenotypes and corresponding sensory profiles of extruded snack samples.

Sample	Instrumental Texture Signature	Dominant Sensory Profile	Oral-Processing Interpretation
0% Okara	Moderate force resistance; moderate acoustic activity; intermediate friction	Moderately crunchy, moderate sound intensity, low hardness, low Coarseness	Starch-dominated brittle fracture with partial lubrication during chewing
29% Okara	Increased force resistance; reduced acoustic emission; elevated friction	Reduced crunchiness, lower sound intensity; increased hardness; slight Coarseness	Reinforced matrix with damped fracture behavior and cohesive bolus formation
40% Okara	High force resistance; weak acoustic emission; high friction with limited lubrication	Low crunchiness and sound intensity, high hardness, pronounced Coarseness	Dense, fiber-rich structure with progressive deformation and resistant bolus formation
NuMunchees	Moderate force resistance; high acoustic activity; moderate lubrication	Crunchy and noisy with moderate hardness	Expanded, brittle structure with sustained fracture events
SnackJack	Intermediate force resistance; moderate acoustic emission; moderate friction	Balanced crunchiness and hardness	Mixed fracture–lubrication behavior with intermediate structural density
Cheetos	Low force resistance; high acoustic intensity; low friction	Very crunchy, loud, smooth mouthfeel	Highly aerated, brittle structure with rapid crack propagation and early lubrication

## Data Availability

The original contributions presented in the study are included in the article, further inquiries can be directed to the corresponding author.

## Supplementary Materials

The following supporting information can be downloaded at: https://www.mdpi.com/article/10.3390/foods15040758/s1, Supplementary File S1: Mechanical–Acoustic Signal Processing Scripts and Extracted Parameters; Table S1: Mechanical–Acoustic parameters extracted from force–acoustic profiles.
