# Supplementary material for: Texture Phenotypes of Fiber-Enriched Extruded Snacks Revealed by Mechanical–Acoustic Analysis, Tribology, and Sensory Mapping
Source: Foods. 2026 Feb 19;15(4):758. doi: 10.3390/foods15040758 (PMC12939363; doi:10.3390/foods15040758)
Supplement: Supplementary file 1 [file foods-15-00758-s001.zip › foods-4097463-supplementary.pdf]

Supplementary File S1. Mechanical–Acoustic Signal Processing Scripts and Extracted Parameters Used in This Study

S1.1: R script for SNR-enhanced acoustic envelope visualization used for mechanical–acoustic signal inspection

```
# ===============================================================
# SNR-enhanced force–acoustic envelope (visualization)
# For Stable Micro Systems exports (3PB and 5AK)
# Output: dual-axis plot (force and acoustic envelope) for visualization only
# ===============================================================

# install.packages(c("readxl", "signal", "zoo", "ggplot2"))
library(readxl)
library(signal)
library(zoo)
library(ggplot2)

# -------------------------
# User input
# -------------------------
file_path <- "29%Okara rep2.xlsx"

# Column names as exported by Stable Micro Systems
col_time   <- "Time (sec)"
col_force  <- "Force (g)"
col_acoust <- "Auxiliary"     # acoustic channel (envelope/raw auxiliary)

# Fixed parameters
hp_order  <- 3
hp_cutoff <- 50      # Hz
env_win   <- 25      # samples (moving-average envelope)
force_win <- 11      # samples (visual smoothing only)

# -------------------------
# Load data
# -------------------------
dat <- read_excel(file_path)

time   <- as.numeric(dat[[col_time]])
force  <- as.numeric(dat[[col_force]])
ac_raw <- as.numeric(dat[[col_acoust]])

# Sampling frequency
dt <- median(diff(time), na.rm = TRUE)
fs <- 1 / dt

# High-pass filtering
Wn <- hp_cutoff / (fs / 2)
bf <- butter(hp_order, Wn, type = "high")
ac_hp <- filtfilt(bf, ac_raw)

# Envelope extraction
env   <- abs(ac_hp)
env_s <- rollmean(env, k = env_win, fill = NA, align = "center")

# Force smoothing (visualization only)
force_s <- rollmean(force, k = force_win, fill = NA, align = "center")
```

```
# Prepare plot dataframe
df <- data.frame(Time = time, Force = force_s, Envelope = env_s)

# Scale envelope for dual-axis plotting
force_max <- max(df$Force, na.rm = TRUE)
env_max   <- max(df$Envelope, na.rm = TRUE)
scale_fac <- force_max / env_max
df$Envelope_scaled <- df$Envelope * scale_fac

# Plot
p <- ggplot(df, aes(x = Time)) +
  geom_line(aes(y = Force), linewidth = 0.7) +
  geom_line(aes(y = Envelope_scaled), linewidth = 0.6) +
  scale_y_continuous(
    name = "Force (g)",
    sec.axis = sec_axis(~ . / scale_fac, name = "Acoustic envelope (a.u.)")
  ) +
  labs(x = "Time (s)", title = "Force–time profile and SNR-enhanced acoustic envelope") +
  theme_minimal(base_size = 11)

print(p)

ggsave("SNR_envelope.png", p, width = 10, height = 4, dpi = 300)
```

S1.2. Mechanical–Acoustic parameters extracted from force–acoustic profiles

Table S1. Mechanical–Acoustic parameters extracted from force–acoustic profiles used for multivariate and index-based analyses.

No.	Parameter name	Unit	Definition
**1**	Baseline Force Mean	g	Mean force in the pre-onset region ($t < 0$), where time is zeroed at force onset; pre-contact force level/instrument noise.
**2**	Baseline Force SD	g	Standard deviation of force in the pre-onset region ($t < 0$).
**3**	Baseline Acoustic Mean	a.u.	Mean acoustic signal in the pre-onset region ($t < 0$); background acoustic noise.
**4**	Baseline Acoustic SD	a.u.	Standard deviation of acoustic signal in the pre-onset region ($t < 0$); used for event thresholding.
**5**	Max Force	g	Maximum force recorded after force onset.
**6**	Time Max Force	s	Time (relative to force onset) at which Max Force occurs.
**7**	Work	g·s	Area under the force–time curve from force onset to the end of the test.
**8**	Stiffness	g/s	Mean positive dF/dt from force onset to the time force first reaches 50% of Max Force.
**9**	Max Acoustic	a.u.	Maximum acoustic amplitude recorded after force onset.
**10**	Time Max Acoustic	s	Time (relative to force onset) at which Max Acoustic occurs.
**11**	Event Count	–	Number of acoustic events detected after force onset using threshold = baseline acoustic mean + 3 SD.
**12**	First Event	s	Time (relative to force onset) of the first detected acoustic event (0 if no events detected).
**13**	Last Event	s	Time (relative to force onset) of the last detected acoustic event (0 if no events detected).
**14**	Duration	s	Acoustic activity duration = Last Event − First Event (0 if no events detected).
**15**	Density	events/s	Event density = Event Count / Duration (0 if Duration = 0).
**16**	Acoustic Energy	a.u.·s	Integral of baseline-corrected acoustic signal, $\int \max(\text{Acoustic} - \text{baseline mean}, 0)\,dt$, after force onset.
**17**	Mean Force at Events	g	Mean force at event-peak time points (0 if no events detected).
**18**	Mech–Ac Ratio	a.u./g	Acoustic Energy / Work.
**19**	Peak Time Diff	s	Absolute time difference between maximum force and maximum acoustic event (|Time Max Force − Time Max Acoustic|).

Notes (Table S1).
(i) **Time reference:** all time-based parameters are reported relative to force onset ($t = 0$).
(ii) **Event detection:** acoustic threshold = baseline mean + 3×baseline SD.
(iii) **No-event handling:** when no acoustic events are detected, event-based parameters are set to 0 to retain observations for multivariate analysis.
(iv) **Direct signal processing:** parameters are computed from exported force–acoustic time series (not dependent on proprietary macros).

S1.3. R script for mechanical–acoustic signal processing and parameter extraction

```
# ============================================================
# Mechanical–Acoustic Signal Processing & Parameter Extraction
# Protocol aligned with Section 2.3.3 of the main manuscript
# All thresholds and parameter definitions are fixed and applied identically across samples
# - Force onset: first time Force > 5 g (t = 0)
# - Baseline: all points before onset (t < 0)
# - Stiffness: mean positive dF/dt from onset to 50% of Fmax
# - Acoustic events: baseline_mean + 3*baseline_sd
#   Event count = number of contiguous above-threshold segments
# - Acoustic energy: integral of pmax(Acoustic - baseline_mean, 0)
# - No-event handling: event-based metrics set to 0
# ============================================================

suppressPackageStartupMessages({
  library(readxl)
  library(dplyr)
})

trapz <- function(x, y) {
  if (length(x) < 2) return(NA_real_)
  sum(diff(x) * (head(y, -1) + tail(y, -1)) / 2, na.rm = TRUE)
}

extract_mech_acoustic <- function(path,
                                  sheet = 1,
                                  time_col  = "Time (sec)",
                                  force_col = "Force (g)",
                                  ac_col    = "Auxiliary",
                                  force_onset_g = 5,
                                  stiff_frac_fmax = 0.50,
                                  ac_k_sd = 3) {

  dat <- read_excel(path, sheet = sheet) %>%
    transmute(
      Time     = as.numeric(.data[[time_col]]),
      Force    = as.numeric(.data[[force_col]]),
      Acoustic = as.numeric(.data[[ac_col]])
    ) %>%
    filter(is.finite(Time), is.finite(Force), is.finite(Acoustic)) %>%
    arrange(Time)

  # ---- Force onset ----
  onset_idx <- which(dat$Force > force_onset_g)[1]
  if (is.na(onset_idx)) stop("No force onset found (Force never exceeded threshold).")

  t0 <- dat$Time[onset_idx]
  dat <- dat %>% mutate(t_rel = Time - t0)

  baseline <- dat %>% filter(t_rel < 0)
  post     <- dat %>% filter(t_rel >= 0)

  # ---- Baseline stats ----
  bF_mean <- mean(baseline$Force, na.rm = TRUE)
  bF_sd   <- sd(baseline$Force, na.rm = TRUE)
  bA_mean <- mean(baseline$Acoustic, na.rm = TRUE)
  bA_sd   <- sd(baseline$Acoustic, na.rm = TRUE)
```

```
# ---- Mechanical metrics ----
Fmax <- max(post$Force, na.rm = TRUE)
t_Fmax <- post$t_rel[which.max(post$Force)]
Work <- trapz(post$t_rel, post$Force)

# Stiffness: mean positive dF/dt from onset to 50% of Fmax
F_stiff_lim <- stiff_frac_fmax * Fmax
stiff_seg <- post %>% filter(Force <= F_stiff_lim)

stiffness <- 0
if (nrow(stiff_seg) >= 3) {
  dF <- diff(stiff_seg$Force)
  dt <- diff(stiff_seg$t_rel)
  slope <- dF / dt
  slope_pos <- slope[is.finite(slope) & slope > 0]
  stiffness <- if (length(slope_pos) > 0) mean(slope_pos) else 0
}

# ---- Acoustic events ----
ac_thr <- bA_mean + ac_k_sd * bA_sd
above <- post$Acoustic > ac_thr

starts <- which(above & !dplyr::lag(above, default = FALSE))
ends   <- which(above & !dplyr::lead(above, default = FALSE))
event_count <- length(starts)

if (event_count > 0) {
  event_times <- numeric(event_count)
  for (i in seq_len(event_count)) {
    seg_idx <- starts[i]:ends[i]
    seg_peak_rel <- seg_idx[which.max(post$Acoustic[seg_idx])]
    event_times[i] <- post$t_rel[seg_peak_rel]
  }

  first_event <- min(event_times)
  last_event  <- max(event_times)
  duration    <- last_event - first_event
  density     <- if (duration > 0) event_count / duration else 0

  event_force <- sapply(event_times, function(te) {
    j <- which.min(abs(post$t_rel - te))
    post$Force[j]
  })
  mean_force_events <- mean(event_force, na.rm = TRUE)

} else {
  first_event <- 0
  last_event  <- 0
  duration    <- 0
  density     <- 0
  mean_force_events <- 0
}

# ---- Acoustic maxima & energy ----
Amax <- max(post$Acoustic, na.rm = TRUE)
t_Amax <- post$t_rel[which.max(post$Acoustic)]

ac_corr_pos <- pmax(post$Acoustic - bA_mean, 0)
```

```r
  acoustic_energy <- trapz(post$t_rel, ac_corr_pos)

  mech_ac_ratio <- if (is.finite(Work) && Work != 0) acoustic_energy / Work else NA_real_
  peak_time_diff <- abs(t_Fmax - t_Amax)

  tibble(
    file = basename(path),
    Baseline_Force_Mean_g = bF_mean,
    Baseline_Force_SD_g   = bF_sd,
    Baseline_Acoustic_Mean = bA_mean,
    Baseline_Acoustic_SD   = bA_sd,
    Max_Force_g = Fmax,
    Time_Max_Force_s = t_Fmax,
    Work_gs = Work,
    Stiffness_g_per_s = stiffness,
    Max_Acoustic = Amax,
    Time_Max_Acoustic_s = t_Amax,
    Event_Count = event_count,
    First_Event_s = first_event,
    Last_Event_s  = last_event,
    Duration_s    = duration,
    Density_events_per_s = density,
    Acoustic_Energy = acoustic_energy,
    Mean_Force_at_Events_g = mean_force_events,
    Mech_Ac_Ratio = mech_ac_ratio,
    Peak_Time_Diff_s = peak_time_diff
  )
}

# ----------------------------
# Example: run on one file
# ----------------------------
res <- extract_mech_acoustic("29%Okara rep2.xlsx")
print(res)

# ----------------------------
# Batch processing example
# ----------------------------
# files <- list.files(".", pattern="\\.xlsx$", full.names=TRUE)
# out <- dplyr::bind_rows(lapply(files, extract_mech_acoustic))
# write.csv(out, "mech_acoustic_extracted_params.csv", row.names = FALSE)
```

S1.4. R script for Stribeck curve smoothing and lubrication regime transition detection

```
# Load required libraries
library(ggplot2)
library(dplyr)

# === 1. Load Raw Data ===
# Replace with actual file path or input method
# Expecting a dataframe with columns: speed_mm_s, friction
raw_data <- read.csv("your_data.csv")

# === 2. Compute U*eta0 (use constant eta0 if needed) ===
# For simplicity, assume η0 = 1 mPa·s here; update as needed
eta0 <- 1e-3  # Pa·s
raw_data <- raw_data %>%
  mutate(U_eta0 = speed_mm_s * eta0)

# === 3. Log–log Transform ===
raw_data <- raw_data %>%
  mutate(logU = log10(U_eta0),
         logF = log10(friction))

# === 4. Apply LOWESS smoothing (locally weighted scatterplot smoothing) ===
loess_fit <- loess(logF ~ logU, data = raw_data, span = 0.2)
smoothed <- data.frame(
  logU = raw_data$logU,
  logF_smooth = predict(loess_fit)
)

# === 5. Compute Derivative ===
smoothed <- smoothed %>%
  mutate(slope = c(NA, diff(logF_smooth) / diff(logU)))

# === 6. Identify Transition Points ===
# 6.1 Boundary→Mixed transition: first sustained slope < −0.05 in log–log space
threshold <- -0.05
b2m_index <- which(smoothed$slope < threshold)[1]
U_b2m <- 10^(smoothed$logU[b2m_index])

# 6.2 Mixed→Hydrodynamic: Global min of smoothed friction
min_index <- which.min(smoothed$logF_smooth)
U_m2h <- 10^(smoothed$logU[min_index])

# === 7. Output Transition Values ===
cat("Boundary → Mixed Uη0:", U_b2m, "\n")
cat("Mixed → Hydrodynamic Uη0:", U_m2h, "\n")

# === 8. Plot (Optional) ===
ggplot() +
  geom_point(data = raw_data, aes(x = logU, y = logF), alpha = 0.3) +
  geom_line(data = smoothed, aes(x = logU, y = logF_smooth), color = "blue") +
  geom_vline(xintercept = log10(U_b2m), linetype = "dashed", color = "red") +
  geom_vline(xintercept = log10(U_m2h), linetype = "dashed", color = "green") +
  labs(x = "log(Uη0)", y = "log(friction coefficient)",
       title = "Stribeck Curve with Lubrication Regime Transitions") +
  theme_minimal()
```

All scripts provided in Supplementary File S1 were used for data processing and visualization in this study and are supplied to ensure analytical transparency and reproducibility. Minor file paths and input names may be adapted by users as needed without affecting the underlying analytical logic.
